# A Regulatory Effect of INMAP on Centromere Proteins: Antisense *INMAP* Induces CENP-B Variation and Centromeric Halo

**DOI:** 10.1371/journal.pone.0091937

**Published:** 2014-03-14

**Authors:** Tan Tan, Zhe Chen, Yan Lei, Yan Zhu, Qianjin Liang

**Affiliations:** 1 Key Laboratory of Cell Proliferation and Regulation Biology of Ministry of Education, College of Life Sciences, Beijing Normal University, Beijing, China; 2 Beijing Key Laboratory of Gene Resource and Molecular Development / Beijing Key Laboratory of Gene Engineering Drugs & Biological Technology, Beijing Normal University, Beijing, China; Tel-Aviv University, Israel

## Abstract

CENP-B is a highly conserved protein that facilitates the assembly of specific centromere structures both in interphase nuclei and on mitotic chromosomes. INMAP is a conserved protein that localizes at nucleus in interphase cells and at mitotic apparatus in mitotic cells. Our previous results showed that *INMAP* over-expression leads to spindle defects, mitotic arrest and formation of polycentrosomal and multinuclear cells, indicating that INMAP may modulate the function of (a) key protein(s) in mitotic apparatus. In this study, we demonstrate that INMAP interacts with CENP-B and promotes cleavage of the N-terminal DNA binding domain from CENP-B. The cleaved CENP-B cannot associate with centromeres and thus lose its centromere-related functions. Consistent with these results, CENP-B in *INMAP* knockdown cells becomes more diffused around kinetochores. Although *INMAP* knockdown cells do not exhibit gross defects in mitotic spindle formation, these cells go through mitosis, especially prophase and metaphase, with different relative timing, indicating subtle abnormality. These results identify INMAP as a model regulator of CENP-B and support the notion that INMAP regulates mitosis through modulating CENP-B-mediated centromere organization.

## Introduction

Normal cell proliferation depends on a whole intact spindle because the accurate segregation of the replicated genome from paired sister chromatids requires the biorientation of chromosomes on the spindle. Errors in the choreography of these processes can lead to aneuploidy or genomic instability, leading to cell death or disease [1], [2]. Centromeres are key chromosomal structures responsible for the correct distribution and assortment of the newly replicated chromosomes from parent cells to daughter cells, and they regulate the chromosome traction dynamics of the spindle during mitosis and meiosis [3]–[5]. After all chromosomes have converged onto the equatorial plate, each pair of sister chromatids is separated and pulled to the opposite poles of the mitotic cell [6].

A three-layer physical structure on centromeres, the kinetochore, which includes inner, middle and transient (outer) domains (plates), contains more than 80 types of proteins [7], [8]. Mitosin/CENP-F is located in the outer plate [9], [10], and CENP-E is localized primarily to the fibrous corona of kinetochores during prometaphase and metaphase [11]. CENP-E and Mitosin/CENP-F play significant roles in kinetochore attachment to spindle microtubules [12]–[14]. There are other spindle checkpoint proteins, such as MAD1, MAD2, BUBR1 and CDC20, in the outer plate [15], [16]. Furthermore, checkpoint control proteins also involve other CENPs, such as CENP-A and CENP-B, as both Poly (ADP-ribose) polymerase 1 (PARP-1) and Poly (ADP-ribose) polymerase 2 (PARP-2) can interact with CENP-A, CENP-B and Bub 3, a protein that plays a role in the inhibition of anaphase-promoting complex or cyclosome (APC/C) by forming a complex with BUB1 under the condition of spindle-assembly checkpoint activation [17], [18]. These findings imply that certain CENPs act as guardians to ensure normal cell division.

Previously, CENP-A, CENP-B and CENP-C were together defined as the pre-kinetochore complex on which the kinetochores assembled in human cells [19]. CENP-B is a highly conserved centromere protein, specifically located at the middle domain of the centromere. It has a DNA binding domain (DBD) within the N-terminal 125-amino acid residue region [20] that binds to 17-bp chromatin DNA repeat units, CENP-B boxes, which widely appear in α-satellite repeats (171 bp each) within human centromeric and pericentromeric DNA sequences [19], [21], [22]. CENP-B and another constitutive centromere protein, CENP-A, are spatially very close (<10 nm) to each other, and CENP-A can serve as a centromeric specialized histone 3 variant, forming a heterodimer with histone H4 in mammalian cells [23]. CENP-C and CENP-T/W also have DNA binding features [24], [25]. CENP-E only localizes to active centromeres and whose knockout is embryonically lethal with disorganized/disordered chromosome segregation [26]. Recent research has shown that CENP-C and CENP-T/W, not CENP-A and CENP-B, act as a type of mediator between the centromeric chromatin platform (constitutive centromere-associated network) and the Knl1-Mis12-Ndc80 complex network, which is beneficial in constructing a bridge between the inner and outer kinetochore [27]–[29]. Up to date, there is limited evidence of direct interaction between CENP-A and any of other CENPs [30], and however CENP-B can interact with CENP-C. These data imply that CENP-B most likely participates in more subtle activities than simple kinetochore assembly.

The absence of CENP-B in cells does not distinctly affect cell division [31], while CENP-B-null mice appear normal but have lower body and testis weights for at least 10 weeks [32]. CENP-A and CENP-B expressions correlate with a clinical phenotype of systemic sclerosis [33]. This implies CENP-B has pivotal functions, as was found over ten years ago, for cell growth and chromosome segregation [34], but may somehow generate a slow influence on cellular and developmental physiology.

INCENP (inner centromere protein), one significant component of CPC (chromosomal passenger complex) [35], contributes to correct chromosome segregation by regulating kinases and spindle assembly [36], [37]. In CPC, INCENP functions as a scaffold-like component, regulating Aurora B kinase and Survivin (an apoptosis inhibitor) [38]. In this complex, Aurora B kinase, the catalytic subunit of CPC, acts as a sensor for the status of attachment of kinetochores to microtubules. Attachment correction and mitotic checkpoint activation are elicited when the phosphorylation of Aurora B targets erroneously attached kinetochores [39]. Recently, researchers have suggested that INCENP also acts as a platform for kinase crosstalk at centromeres, which enables Polo (a key cell cycle kinase that promotes stable microtubule attachment) and Aurora B (a detachment promoter) to achieve a balance in correcting the mis-attachment of microtubules [40].

Inevitably, the functional regulation of the centromere/kinetochore intermolecular relationship and especially the related proteins' modification influences cellular life broadly. Abnormal cell cycle progression is always related to abnormal mitotic apparatus. Disruption of some mitotic regulators causes drastic phenomena while others show mild effects in the cells. For instance, *CENP-H* RNAi cells do not show mitotic arrest, although chromosomes align aberrantly [41]. The CENP-B deficient mice appear normal, but their reproduction is dysfunctional with abnormal sexual organ weight. Knockout also causes age-dependent reproductive deterioration in R1 and W9.5 *Cenpb* null females and complete reproduction incompetence in C57 background *Cenpb* null mice [32]. These findings imply that some gene deficiencies that only display mild cellular changes may lead to hysteretic effects on growth or development.

INMAP is an interphase nucleus/mitotic apparatus protein involved in spindle formation and cell cycle progress we discovered a few years ago. Its over-expression in HeLa cells leads to spindle defects, mitotic arrest and polycentrosome/multinucleus formation with growth inhibition [42], but a high level of INMAP in normal cells represses the gene transcription of tumor suppressor p53 and transcription activator AF-1 [43]. However, how cell growth will be affected by *INMAP* knockdown or what will happen afterward remains undetermined. To further explore the function of INMAP and identify the linkage between this important spindle protein and centromere structure/function, we used the Tet-off system and analyzed certain centromere proteins through immunofluorescence and immunoblotting techniques.

## Materials and Methods

### Cell culture, reagents and antibodies

HeLa cells were cultured in Dulbecco's modified Eagle's medium (DMEM) and RPMI-1640 Medium (Invitrogen, USA) containing 10% (v/v) fetal calf serum (FCS) (Invitrogen, New Zealand) at 37 °C in the presence of 5% CO_2_. DAPI (4', 6-diamidino-2-phenylindole), penicillin and streptomycin were purchased from Sigma, USA. Anti-INMAP monoclonal antibody was obtained as previously described [42]. Other reagents included the high-efficiency transfection reagent Translipid (TransGen Biotech, China), Hygromycin (Roche, USA) and anti-INCENP (Abcam, USA), anti-CENP-B C-terminus (Abcam, USA), anti-α-tubulin (Santa Cruz, USA) and anti-Flag (MBL, Japan) antibodies. Alkaline phosphatase-conjugated goat anti-rabbit IgG, goat anti-mouse IgG antibodies TRITC- conjugated goat anti-rabbit IgG and FITC-conjugated goat anti-mouse IgG were obtained from Vector Laboratories (Peterborough, UK). Oligonucleotides were synthesized by Sangon (Shanghai Sangon Biotechnology, China).

### Plasmid construction and transfection of HeLa cells

pTRE-INMAP (-) was generated by PCR using pEGFP-C3-INMAP as a template with a 5′ primer ATTTGCGGCCGCTGAATGGGGCCCATGTTG and a 3′ primer CGGGATCCTTCATTGTACTTGGACAG and then cloned into pTRE-HA (Clontech, USA). The PCR reaction profile was 94 °C for 5 min followed by 31 cycles of 94°C for 30 s, 55°C for 30 s and 72°C for 1 min, with a final extension at 72°C for 10 min. The selective marker gene *Hygromycin* was obtained from pTRE2hyg vector with *Xho*Idigestion; pTRE-INMAP (-) was also digested with *Xho*I and dephosphorylated with CIAP (Promega, USA). The *Hygromycin* gene and pTRE-INMAP (-) were ligated with DNA ligase (Fermentas, USA). *Hygromycin* in Tet-Off-INMAP cells was detected using a 5′ primer, TGTCCTGCGGGTAAATAGCT, and a 3′ primer, AATAAGGGCGACACGGAAAT. The recombinant vector pTRE-INMAP (-) was transfected into HeLa cells by using high efficiency transfection reagent Translipid according to the manufacturer's instructions (TransGen Biotech, China). Stably transfected cells were selected with 600 ng/ml G418 (Geneticin) for 14 d, and the conditional *INMAP* silenced cell lines were established. Expression vectors for p3XFlag-cmv-9-CENP-B and p3XFlag-cmv-9-CENP-BCT (CENP-B C-terminus variant absent of the aa 1∼135 region) were constructed by PCR amplification with the primers 5′ GGAATTCGGGTCCCAAGAGACGACAG 3′ and 5′ GCTCTAGATCAGCTTTGATGACCAAGACC 3′ and primers 5′ GGAATTCCAGCGGAGTGGCCCG 3′ and 5′ GCTCTAGATCAGCTTTGATGACCAAGACC 3′, respectively. The p3XFlag-cmv-9 vector was purchased from Sigma Aldrich, USA. The recombinant vectors were transiently transfected into HeLa cells. Twenty-four hours after transfection, cells were incubated with antibodies and DAPI stained before observing them with an Olympus laser-scanning confocal microscope (Olympus Fluoview FV300, Japan).

### MTT assay

Four thousand cells were seeded in every well of tissue culture plates. According to the time course, 5 mg/ml MTT (3-[4,5-dimethylthiazol-2-yl]-2,5-diphenyltetrazolium bromide) was added into each well and incubated with the cells for 4 h at 37 °C; then 100 μl lysis solution (10% SDS +0.1% NH_4_Cl) was added into each well and incubated for 4 h or overnight at 37 °C to solubilize the formazan products. Absorbance values were measured at 570 nm with a 550 microplate reader (BIO-RAD, USA). Blank values, indicating the absorbance of MTT and lysate solution only, were subtracted from all samples.

### Giemsa's staining

Cells were rinsed with PBS three times, fixed in 100% methanol for 30 minutes and washed in tap water. A fresh solution of 10% Giemsa stain was prepared in distilled water. Cells were stained for 30 minutes in the dark before rinsing with tap water and thoroughly drying using blotting paper.

### Western blot analysis

Total protein extracts of HeLa cells or Tet-off HeLa cells were loaded onto 12% gels, separated by SDS-PAGE, and transferred onto a nitrocellulose membrane for 3 h at 300 mA. The membrane with transferred polypeptides was immersed in PBST (phosphate-buffered saline (PBS) containing 0.1% Tween 20) with 5% skim milk at room temperature (RT) for 1 h and then probed with monoclonal antibodies according to manufacturers' protocols. After washing three times with PBST, the membranes were incubated with peroxidase-conjugated secondary antibodies for 1 h, and then proteins were detected using an ECL chemiluminescence kit and Kodak BioMix films.

### Indirect immunofluorescence

Cells grown on coverslips were washed with PBS three times and fixed with 1.6% paraformaldehyde at RT for 10 min, followed by permeabilization with 0.1% Triton X-100 at RT for 10 min. After washing another three times with PBS containing 0.1% Triton X-100 and 20 mM glycine and blocking with 5% bovine serum albumin (BSA) in PBS for 10 min at 37°C, the coverslips were incubated with primary antibodies for 1 h at 37°C. The cells were then washed another three times (5 min each) in PBST and incubated for 1 h at 37°C with fluorescein isothiocyanate (FITC)-conjugated goat anti-mouse IgG (1∶50) or goat anti-rabbit IgG (1∶50). These cells were washed again three times with PBST and counterstained with DAPI.

### Microscopic and image analysis

Images of fluorescent cell nuclei were acquired with a ZEISS Laser Scanning Confocal Microscope LSM700 (ZEISS, Germany) equipped with a 40× Plan-Neofluar oil immersion lens (NA = 1.30). Laser power was adjusted to maximize the dynamic range of each sample. For multiple-staining samples, the adjustable spectral window of fluorescence collection was set for each channel with a singly colored control sample so that cross-contamination among channels was avoided. Statistical analyses were performed by the Shapiro-Wilk test and the independent-samples T test using SPSS 16.0 software.

### His-Tagged protein expression and Pulldown

*INMAP* was cloned into the pET30a (+) vector (Invitrogen, USA). The resulting construct was transformed into the bacterial strain BL21 (TransGen Biotech, China) for expression. His-tagged protein was purified on Ni^2+^ beads (GE, USA) from BL21 lysates. For His Pulldown assays, Ni^2+^ beads with INMAP were incubated with HeLa cell extract for 2 h, isolated by centrifugation, washed, and eluted by boiling in Laemmli sample buffer.

### Immunoprecipitation

After HeLa cells were transfected transiently for 24 h, cells were washed with PBS and lysed in RIPA buffer (50 mM Tris, pH 7.5, 150 mM NaCl, 1 mM EDTA, 0.25% sodium deoxycholate, 1% NP-40) containing protease inhibitors (0.1 mg/ml aprotinin, 10 μg/ml each of leupeptin, chymostatin and pepstatin) for 5 min on ice. Protein extracts were clarified by centrifugation for 15 min at 13,000 g at 4 °C. For immunoprecipitation, the extracts were incubated with antibodies against Flag overnight at 4 °C, followed by adding Protein A/G plus-Sepharose (TransGen Biotech, China) and incubating for 4 h at 4°C. After centrifuging for 1 min at 4°C, the pellets were washed five times with 500 μl of cell-lysis buffer while keeping on ice. Washed pellets of protein complexes were resuspended in 40 μl SDS sample buffer, vortexed, boiled, centrifuged for 30 s, and analyzed by SDS-PAGE and Western blotting.

## Results

### INMAP localizes at dot structures in nucleus and interacts with CENP-B

**Figure 1:**
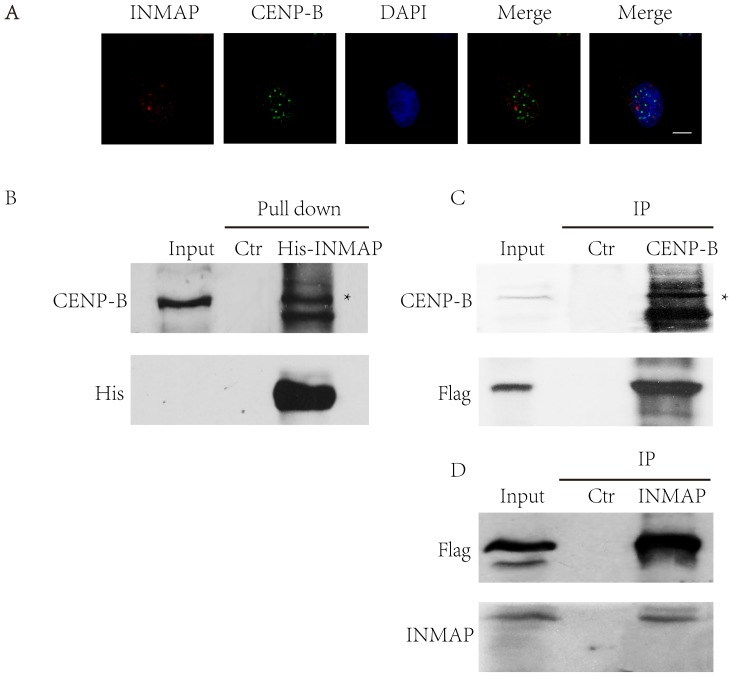
INMAP localizes at dot structures in nucleus and interacts with CENP-B. **A**. Sub-cellular localization of INMAP and CENP-B in HeLa cell nucleus was analyzed with anti-INMAP (red), anti-CENP-B (green) monoclonal antibodies and DAPI (blue). INMAP distributes as a dotted pattern, but the dots almost do not overlap centromeres, marked by the typical centromere protein CENP-B, with some association with a little background CENP-B signal. Bars represent 10 μm. **B**. His-INMAP was added into cell extracts, incubated, and recovered on beads. The Pulldown product was analyzed by Western blotting with anti-CENP-B antibody. A 10% input and a control Pulldown using empty beads were loaded in the left and middle lanes. Asterisk indicates the CENP-B band. **C**. The proteins of HeLa cells that expressed Flag-INMAP was extracted and than incubated with protein A/G with Flag antibody. The immunoprecipitation result was analyzed by Western blotting with anti-CENP-B antibody. An input and a control immunoprecipitation using IgG were loaded in the left and middle lanes. Asterisks indicate the CENP-B bands. **D**. The proteins of HeLa cells that expressed Flag-CENP-B was extracted and than incubated with protein A/G with Flag antibody. The immunoprecipitation result was analyzed by Western blotting with anti-INMAP antibody. An input and a control immunoprecipitation using IgG were loaded in the left and middle lanes.

Our pervious experiments showed that INMAP is located at mitotic apparatus (spindle, centromere and centrosome) in mitotic cells and we also determined that INMAP is located at nucleus in interphase [42] but it is not clear that how INMAP regulates mitosis. In this study, we tested INMAP and CENP-B localization in HeLa cells and found that they can both localize in nucleus and form dot structural pattern (Figure 1A) and in some circumstances INMAP focus overlapped CENP-B (Figure S1), which implied that they may have interaction condition. To test our hypothesis, we found INMAP can interact with CENP-B by Pulldown and immunoprecipitation experiment (Figure 1B, C and D).

### *INMAP* knockdown destabilizes the association of CENP-B with centromeres

**Figure 2:**
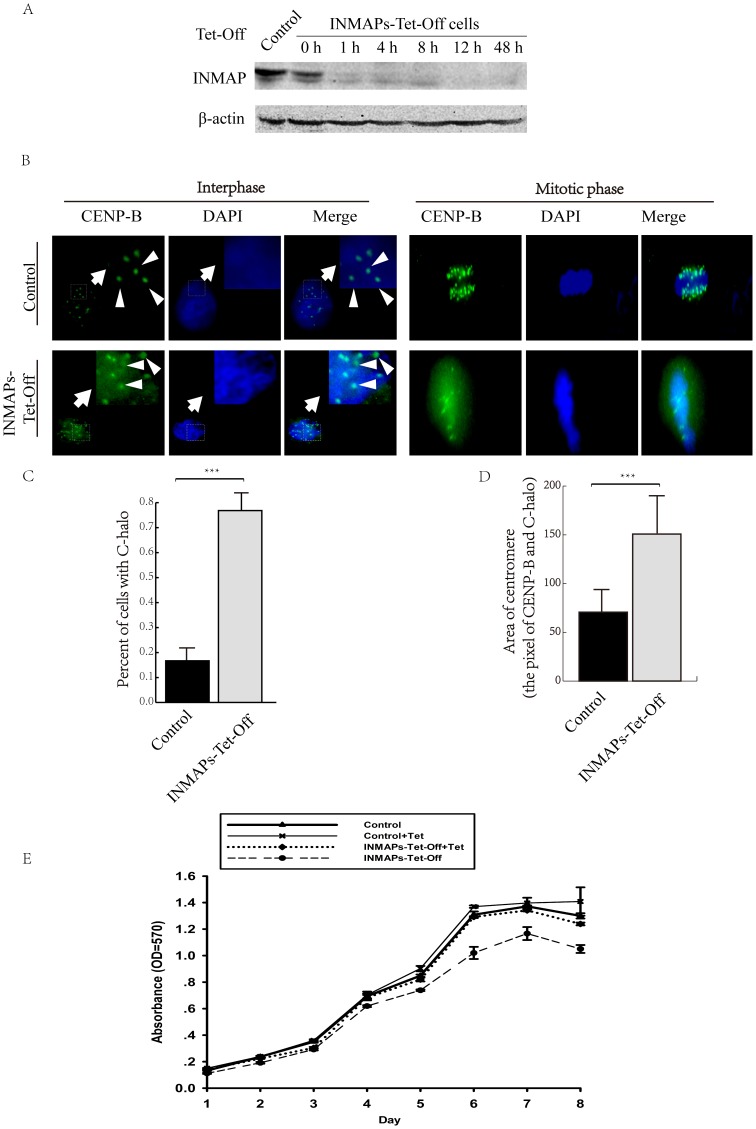
*INMAP* knockdown destabilizes CENP-B association with centromeres. **A**. The selection of INMAPs-Tet-Off cells. The expression quantity of INMAP was detected by immunoblotting in INMAPs-Tet-Off after removing tetracycline. **B**. Centromere structure was analyzed with anti-CENP-B monoclonal antibody (green) and DAPI (blue) in HeLa and INMAPs-Tet-Off cells. INMAPs-Tet-Off cells had a halo like structure around the centromeres (C-halo), more distinctly shown by the amplified pictures in the interphase panels. Bars represent 10 μm. **C**. The statistical analysis of C-haloes in the HeLa and INMAPs-Tet-Off cells. There is a significant difference between HeLa and INMAPs-Tet-Off cells. More than 100 cells were used in statistics, n = 3, ***P<0.001. **D**. The statistical analysis of centromeric CENP-B fluorescent dot (focused fluorescence center plus halo) areas in the HeLa and INMAPs-Tet-Off cells. More than 130 CENP-B fluorescent dots were used in statistics, n = 3, ***P<0.001. **E**. Control and INMAPs-Tet-Off cells were cultured under designated conditions, and the relative cell number was determined by MTT assay.

We constructed a regulatable plasmid pTRE-hyg-INMAP (-) to create INMAPs-Tet-Off cells (HeLa Tet-Off transfected with antisense *INMAP*, which induces *INMAP* silence absent of tetracycline). INMAPs-Tet-Off cells are unable to express *INMAP* without the addition of tetracycline. First, we constructed the pTRE-INMAP (-) regulatable plasmid, amplified the anti-hygromycin gene from pTRE2hyg plasmid and then ligated it to pTRE-INMAP (-). Double enzymatic digestion results showed that pTRE-hyg-INMAP (-) was successfully constructed (Figure S2A). We then transfected pTRE-hyg-INMAP (-) into HeLa cells and selected the INMAPs-Tet-Off cells with hygromycin. We tested the INMAPs-Tet-Off cells by immunoblotting. The *INMAP* was almost completely silenced in 12 h after removal of tetracycline (Figure 2A). Moreover, to validate the INMAPs-Tet-Off cell strain, we tested for *hygromycin* gene by PCR (Figure S2B).

After that, we detected the centromere phenotype reflected by special centromeric components (Figure 2B and Figure S3). We found that INMAPs-Tet-Off cells showed distinct halo-like staining of CENP-B, which we termed centromeric halo (C-halo), as well as a diffuse signal around the nucleus by immunofluorescence. By careful observation of the C-halo, it appears that a portion of CENP-B is neither ‘assembled’ onto the centers of centromeres nor migrates far away from centromeres, while another portion scatters all over the interphase nucleus/mitotic cytoplasm (Figure 2B). We performed statistical analysis on the C-halo-containing nuclei/cells and normal nuclei/cells of both the ordinary HeLa cells and INMAPs-Tet-Off cells (Figure 2C). The number of C-halo-containing INMAPs-Tet-Off cells was significantly greater than that of ordinary HeLa cells (n = 3, P<0.001). We also compared the area of centromeric CENP-B fluorescent dots (focused fluorescence centers plus haloes) between normal HeLa cells and INMAPs-Tet-Off cells (Figure 2D). The area of the fluorescent dots in INMAPs-Tet-Off cells was wider than that of control as the CENP-B became diffused when *INMAP* knockdown. This phenomenon implies that the centromeres became abnormal and unstable due to the inhibition of *INMAP* but that this change did not result in immediate disaster. There was also no significant difference in the cell morphology between the control and INMAPs-Tet-Off cells after tetracycline removal and culturing for 24 h (Figure S2C).

However, INMAPs-Tet-Off cells grew more slowly than controls, becoming significant after six days by MTT assay (p<0.05, n = 4) (Figure 2E). The result implied that the growth of INMAPs-Tet-Off cells was not immediately inhibited after *INMAP* was knocked down and that the effect of *INMAP* silence was a slow process. On the seventh day, the INMAPs-Tet-Off cells began to grow faster than the controls with the loss of cellular contact inhibition. After the seventh day, living cell numbers of all the groups decreased accompanied by rampant cell death (Figure 2E). From the sixth to seventh day, it seems that INMAPs-Tet-Off cells exhibited contact inhibition despite not reaching the same cell density as the control cells. In other words, INMAPs-Tet-Off cells cannot tolerate the ‘normal’ cell destiny. We think the moderately retarding cell proliferation attributed to the abnormal CENP-B when *INMAP* was inhibited.

### *INMAP* knockdown promotes N-terminal cleavage of CENP-B and CENP-BCT localizes out of nucleus

**Figure 3:**
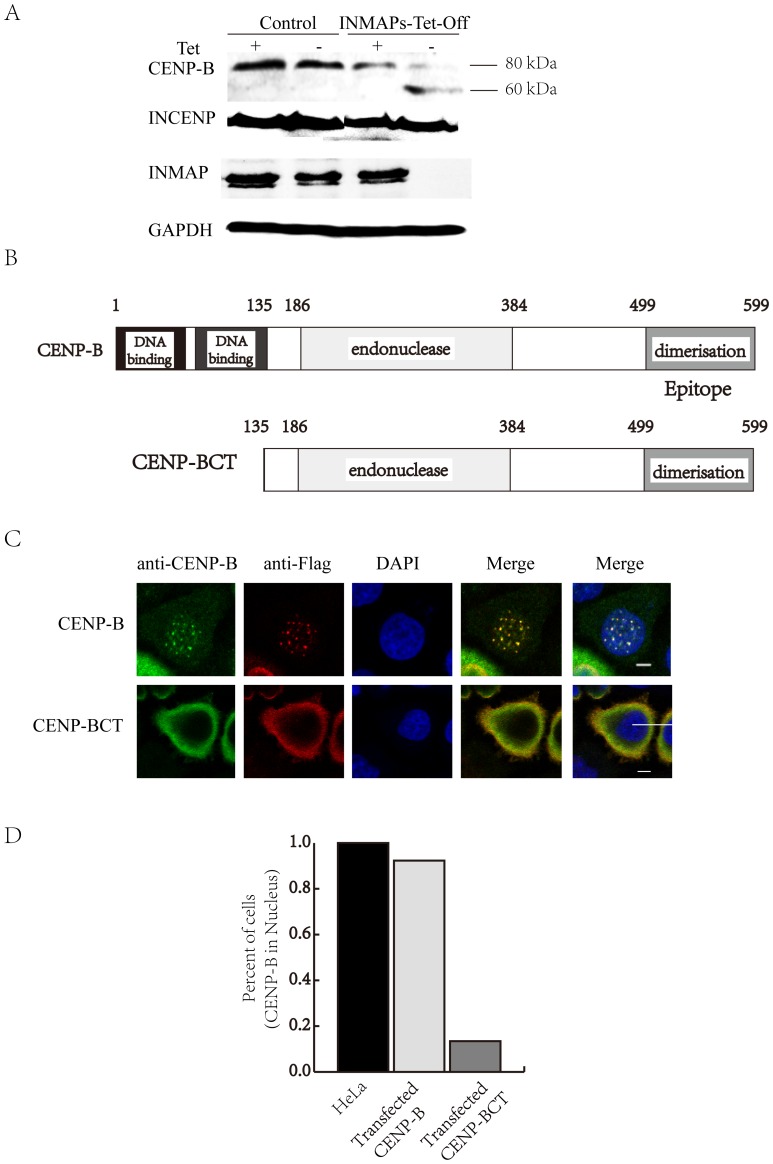
*INMAP* knockdown promotes N-terminal cleavage of CENP-B and CENP-BCT localizes out of nucleus. **A**. The analysis of CENP-B bands in INMAPs-Tet-Off cells (+Tet) and INMAPs-Tet-Off cells (-Tet). It showed 80-kDa and 60-kDa band in INMAPs-Tet-Off cells (-Tet) while other controls did not. **B**. Conserved domains of CENP-B. The epitope of CENP-B antibody was 499–599 amino acids. **C**. Transient transfection of the full-length CENP-B (Flag-CENP-B) and the truncated CENP-B variant lacking the DNA-binding domain and putative DNA-binding domain (Flag-CENP-BCT) in HeLa cells and detected by fluorescence microscope, bars represent 10 μm. **D**. The statistical analysis of the proportion of cells in which CENP-B is located in the nucleus and cytoplasm in HeLa cells and *CENP-B* and *CENP-BCT* transfected cells. More than 200 cells were used in statistical analysis.

To explore the C-halo phenomenon and its significance, we further tested two inner centromeric proteins, INCENP and CENP-B, by immunoblotting (Figure 3A). Interestingly, the INCENP expression level in INMAPs-Tet-Off cells did not display deviation from the control cells, nor did its molecular weight. Unlike INCENP, CENP-B displayed two molecular weight bands: one of 80 kDa, the same as the control, and the other of 60 kDa, a truncated CENP-B protein not detected in the control. To further determine how the CENP-B 60-kDa variant affected the centromere, we analyzed the structure of CENP-B, which has a known DNA-binding domain and a putative DNA-binding domain in its N terminus and a CENP-B dimerization domain in its C terminus (Figure 3B). Because the antibody we used recognizes the C terminus of CENP-B, we can infer that the 20-kDa truncation occurs at the N-terminus (aa1∼135) involving both the known and the putative DNA-binding domains. Thus, we transiently transfected and expressed full-length CENP-B (80 kDa) and a CENP-B variant (CENP-BCT) lacking the 135-aa fragment into HeLa cells to observe the phenotype. Surprisingly, the full-length exogenous CENP-B can co-localize with the endogenous CENP-B on centromeres, while truncated exogenous CENP-B (CENP-BCT) was dispersed out of nucleus. The endogenous CENP-B could not be found in the nucleus both (Figure 3C and S4). We also made the analysis of the proportion of cells with CENP-B locating at nucleus in transfected *CENP-B* (92%), *CENP-BCT* (11.3%) and normal cells (100%) (Figure 3D). Transfected *CENP-BCT* cells significantly had fewer cells with CENP-B in nucleus, and even if CENP-BCT enters nucleus, it can not form the dot structure of centromeres.

### *INMAP* knockdown does not affect the overall structure layout of spindles

**Figure 4:**
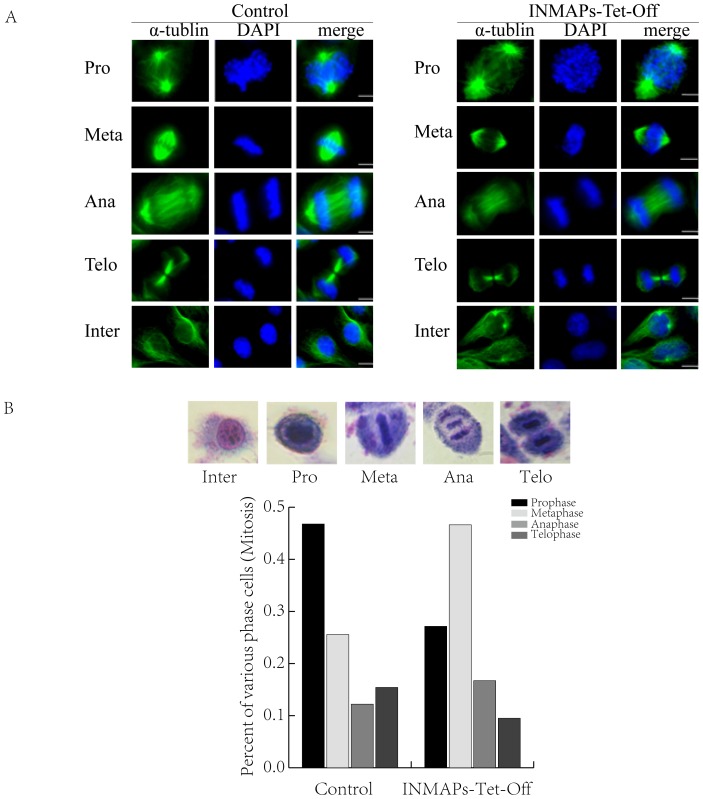
*INMAP* knockdown moderately retards cell proliferation but not affects the overall structure layout of spindles. **A**. Control and INMAPs-Tet-Off cells were cultured in tetracycline-free medium for indicated hours and photographed under a fluorescence microscope. Bars represent 10 μm. **B**. Control and INMAPs-Tet-Off cells were cultured under designated conditions, and the relative cell number was determined by MTT assay. **C**. Control and *INMAP* knockdown cells in prophase, metaphase, anaphase, telophase and anaphase were immunostained with anti-tubulin antibodies and counterstained with DAPI. Bars represent 10 μm. **D**. Top lane: various mitotic phase cells by Giemsa staining. A histogram displays the proportions of various mitotic phase control cells and INMAPs-Tet-Off cells. More than 600 cells were used for statistics.

We also tested spindle status in INMAPs-Tet-Off cells (Figure 4A). However, we did not find any notable abnormality on spindles. We also performed statistical analyses of the various mitotic phases of INMAPs-Tet-Off cells to determine whether *INMAP* silence could affect mitosis. Interestingly, the proportion of the various mitotic phases of INMAPs-Tet-Off cells dramatically changed compared with the control. The quantity of metaphase cells accounted for 46.63%, much more than the prophase (27.15%), anaphase (16.72%) and telophase (9.51%) percentages, respectively.

## Discussion

In the present study, we found INMAP and CENP-B are both localized in nucleus and INMAP can interact with CENP-B (Figure 1). When *INMAP* was over-expressed in HeLa cells, it caused multiple polar spindles [42]. When *INMAP* expression was inhibited in INMAPs-Tet-Off cells, there did not displayed significant difference in cell growth from the control and INMAPs-Tet-Off cells until the sixth day (Figure 2E). INMAP deficiency also affected chromosome separation during mitosis. Specifically, the percentage of INMAPs-Tet-Off cells was lower than the control in prophase while higher in metaphase (Figure 4B). These findings implied that *INMAP* functions importantly and its silence, different from its over-expression [42], shows a retard mode that must be involved in subtle modulation.

Some centromere proteins affect not only the processivity of spindles [44] and the depolymerizing activity of microtubules [45] but also the recovery from spindle damage [46]. As a constitutive centromere protein, human CENP-B connects with α-satellite DNA at CENP-B boxes and maintains the intact centromeres [47]. To explain why the INMAPs-Tet-Off cells displayed a delayed pattern of slowed growth after *INMAP* inhibition, we analyzed their spindles and centromeres. We discovered variation in the CENP-B spatial pattern (Figure 2B) and molecular weight (Figure 3A), though not any dramatic spindle deficiency/disorder was detected by indirect immunofluorescence for α-tubulin (Figure 4A). Specifically, CENP-B could no longer stably localize on centromeres, and instead, it partly encircles centromeres, forming a relatively diffuse and nebula-like structure, centromeric halo/C-halo. With C-halo formed, a portion of relatively diffuse CENP-B collected around the dense CENP-B signal center, as though it had migrated away from the center without being degraded. In the images, it is easy to distinguish the centromeres in INMAPs-Tet-Off cells that ‘swell’ compared to those in control cells. This scene becomes more outstanding when we look at the mitotic phase. Although we can see some CENP-B signal background scattered in the control cells (or in nuclei at interphase), the ‘halo’ is more pronounced in INMAPs-Tet-Off cells. Therefore, it is obvious that CENP-B was affected by *INMAP* silence and reflected the changed centromere organization. This change was accompanied by the molecular weight variation of CENP-B; in INMAPs-Tet-Off cells, *CENP-B* not only expressed the original 80-kDa form but also generated a 60-kDa form (Figure 3A).

CENP-B contains sites that are highly sensitive to proteases, and among them is the one at which the whole molecule can be cleaved by proteolysis such that the DNA binding domain (125 amino acid region from N-terminus) is separable from the dimerizing activity domain (a 20-kDa fragment at the C-terminus) [20]. *INMAP* silence may activate certain proteases through an unknown mechanism to cleave the N-terminal 20-kDa peptide from CENP-B. Because of this cleavage, the CENP-B dimers can form with intact dimerizing domains, while a region of the N-terminus with the DNA binding domain is lost, rendering CENP-B unable to attach to DNA. This hypothesis suggests that the CENP-B-DNA complex become loose and unstable, leading to a C-halo pattern around the centromeres (Figure 2B).

**Figure 5:**
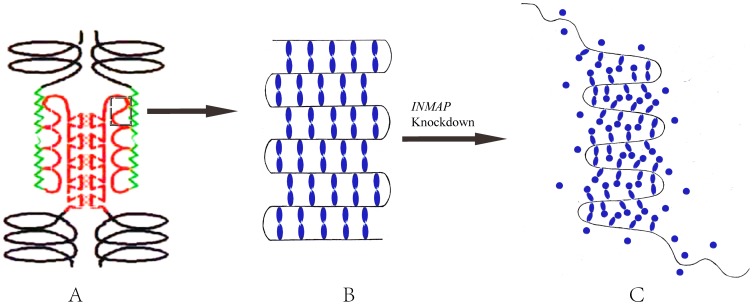
A deductive model delineating the relationship between CENP-B and α-DNA. **A**. The normal structure of the centromere, adapted from He D and Brinkley BR (1997). CENP-B is specifically located at the middle domain of the kinetochore domain of a centromere. It has a DNA binding domain (DBD) within the N-terminal 125 residues that binds to 17-bp CENP-B boxes that widely appear in α-satellite repeats. The black dotted frame represents an area where α-DNA binds with CENPs; the green wavy line represents the outer proteins of the centromere; the red triangles indicate inner structure of the centromere. **B**. Stretched diagrammatic sketch showing the binding of CENP-B dimers to α-DNA at CENP-B boxes. **C**. A model showing the loose connection between CENP-B and α-DNA in the INMAPs-Tet-Off cells, which may illustrate the C-halo pattern. Some CENP-B molecules, symbolized as round patches instead of elliptic ones, cannot bind DNA due to the lack of the 20-kDa fragments at their N termini; some are even free from α-DNA, causing the C-haloes around the centromeres, with partly diffused normal compensatorily expressed CENP-B molecules (not shown).

As far as the C-halo is concerned, it is true that no immediate dramatic events have been implied in INMAPs-Tet-Off cells, but there is no reason to ignore the cytophysiological reaction just because of its tardiness. Let's connect a weather phenomenon in the mind (Figure S5). When a halo appears around the moon, it is likely that the next day will be windy. It is not beyond logic or reason that cells grow in a delayed inhibitory pattern in response to *INMAP* silence. We constructed a model to explain the C-halo phenotype based on the above discussion (Figure 5). The 60-kDa CENP-B variant, lacking the N terminus, led to the C-halo formation. This functional variation may lead to new insights. Previous studies revealed that a 17-bp motif termed ‘CENP-B box’ furnishes alphoid repeat monomer binding sites for CENP-B proteins, which plays a crucial role in the formation of the specified structure and/or function of the centromere [21], and further studies revealed a ‘fold-back’ mode by which CENP-B, with its N-terminal binding domain, associates with chromatin α-DNA at its CENP-B boxes; at C-termini, pairs of CENP-B molecules are dimerized [48]. The relevant studies indicated that tandem 5'-GA:GA-3' mismatches are responsible for the enhanced stability of the fold-back structures formed by the *Drosophila* centromeric dodeca-satellite; other centromeric DNA sequences, such as the AAGAG satellite of *Drosophila* and the mammalian CENP-B box sequence, contribute to the formation of special intramolecular hairpins [49]. CENP-B also functions as a centromeric structural factor for establishing a unique centromere-specific pattern of nucleosome positioning [50]. CENP-B null mice have lower body and testis weights [51], but they are viable with no other apparent abnormalities [52], and interestingly for us, they are mitotically and meiotically normal [51]. As for the molecular forms, we found both 80-kDa CENP-B and its 60-kDa variant in INMAPs-Tet-Off cells, but the level of the former was considerably lower than that of the latter (Figure 3A), while they existed as a larger total quantity in INMAPs-Tet-Off than in control cells. This finding implied that, after *INMAP* silence, the cells are able to generate a certain sum of compensatory CENP-B to overcome the deficit from protein cleavage, in spite of the limitation caused by abnormal gene expression, and as a result, the amount of 80-kDa CENP-B plus its 60-kDa variant in a INMAPs-Tet-Off cell exceeds the total CENP-B in a cell.

Just like the aureoles forming the lunar halo (Figure S5), the centromeric haloes were also accompanied by diffuse centromere-surrounding protein in our experiment because a portion of chromatin was free from CENP-Bs, and the‘idle’CENP-B variants would scatter in the interphase nucleus/mitotic cytoplasm (Figure 5). How silenced *INMAP* contributes to the centromeric structural variation through the truncation of CENP-B and what signaling pathway it functions through are new topics we will explore in the near future.

Although we transiently transfected and expressed a 60-kDa CENP-B (CENP-BCT) lacking the N-terminal DNA binding domain, we did not find the C-halo phenotype in these cells. This result proves that the C-halo caused by *INMAP* silence depends on the cleavage of the preexistence of mature CENP-B proteins in nuclei / centromeres. As the exogenous vector translated a mutant CENP-B lacking the DNA binding domain, it will not necessarily enter nuclei to locate in the centromeres, and we only observed it out of nuclei (Figure 3C and S4).

Researchers revealed that many CENPs, such as CENP-A, Mis12, CENP-C, CENP-H and CENP-I, are all localized in a core domain of the centromeric chromatin [4]. These CENPs, when associated with CENP-B, maintain centromeric stability and function. Moreover, we also tested INCENP in this study and found that *INMAP* silence did not affect the *INCENP* expression or its molecular weight, indicating that the centromere inner structure may be intact to a certain degree, because INCENP is a scaffold-like component in CPC and a platform for kinase crosstalk at centromeres and contributes to achieving a balance in correcting microtubule mis-attachment [40]. This finding can explain why truncated CENP-B and the C-halo phenomenon influenced cells with a delayed growth inhibition mode.

The relationship of INMAP with other centromere structural proteins and the pathway by which it regulates the centromere structure and chromosome separation are questions worth answering. These studies may reveal new insights into mitotic regulation and the CENP-B-mediated centromere organization.

## Supporting Information

Figure S1 **Close correlation between INMAP and CENP-B under a certain circumstances.** Sub-cellular localization of INMAP and CNNP-B in HeLa cell nucleus was analyzed with anti-INMAP (red), anti-CENP-B (green) monoclonal antibodies and DAPI (blue). INMAP focus can overlap centromere in some circumstances, marked by the typical centromere protein CENP-B as the arrows indicated. Bar represents 10 μm.
(TIF)

Figure S2 **pTRE-hyg-INMAP (-) vector construction.** A. The analysis of pTRE-INMAP (-) and pTRE-hyg-INMAP (-) double restriction enzyme digestion. Lane 1: DNA markers. Lane 2: pTRE-hyg-INMAP (-) double restriction enzyme digestion. Lane 3: pTRE-INMAP (-) double restriction enzyme digestion. B. DNA was extracted from INMAPs-Tet-Off cells and *hygromycin* gene was detected by PCR. Lane 1: DNA markers. Lane 2: Amplifying *hygromycin* from the HeLa cell genome by PCR. Lane3: Amplifying *hygromycin* from INMAPs-Tet-Off cells by PCR. C. Control and INMAPs-Tet-Off cells were cultured in tetracycline-free medium for indicated hours and photographed under a phase contrast microscope at 40× objective lens. Bars represent 50 μm.
(TIF)

Figure S3 **INMAPs-Tet-Off cells showed distinct halo-like staining of CENP-B.** Centromere structure was analyzed with anti-CENP-B monoclonal antibody (green) and DAPI (blue) in HeLa and INMAPs-Tet-Off cells. INMAPs-Tet-Off cells had a halo like structure around the centromeres (C-halo). Under the same photographing condition, the clear doted CENP-B signals appeared in control, but “haloes” in the experimental group with diffused background. Bars represent 10 μm.
(TIF)

Figure S4 **Scanning various cellular stacks of CENP-BCT cells with laser scanning confocal microscope.** Transient transfection of the truncated CENP-B variant lacking the DNA-binding domain and putative DNA-binding domain (Flag-CENP-BCT) was conducted in HeLa cells and analyzed with anti-Flag (red), anti-CENP-B (green) monoclonal antibodies and DAPI (blue) by laser scanning confocal microscope, bars represent 10 μm. Interval of two stacks was 1 μm.
(TIF)

Figure S5 **The moon in the sky at a clear night and a cloudy night. C-halo is like the lunar halo in the night.** Left: the moon appears in the sky at a clear night without clouds or wind. A clear outline of the moon can be observed. This situation is analogous to the centromere in the normal nucleus. Right: the moon appears in the sky at a cloudy or windy night. Many halo-like clouds or fog appears near the moon. This situation predicts a rainy or foggy subsequent day. The centromere in the INMAPs-Tet-Off cells is similar to this type of moon, and CENP-B fluorescent signal is similar to the lunar halo around the centromere. The halo also predicts some non-dramatic events in the cell, e.g., the cell grows slowly.
(TIF)
