# Supplementary figures and images for: A Regulatory Effect of INMAP on Centromere Proteins: Antisense INMAP Induces CENP-B Variation and Centromeric Halo
Source: PLoS One. 2014 Mar 14;9(3):e91937. doi: 10.1371/journal.pone.0091937 (PMC3954832; doi:10.1371/journal.pone.0091937)

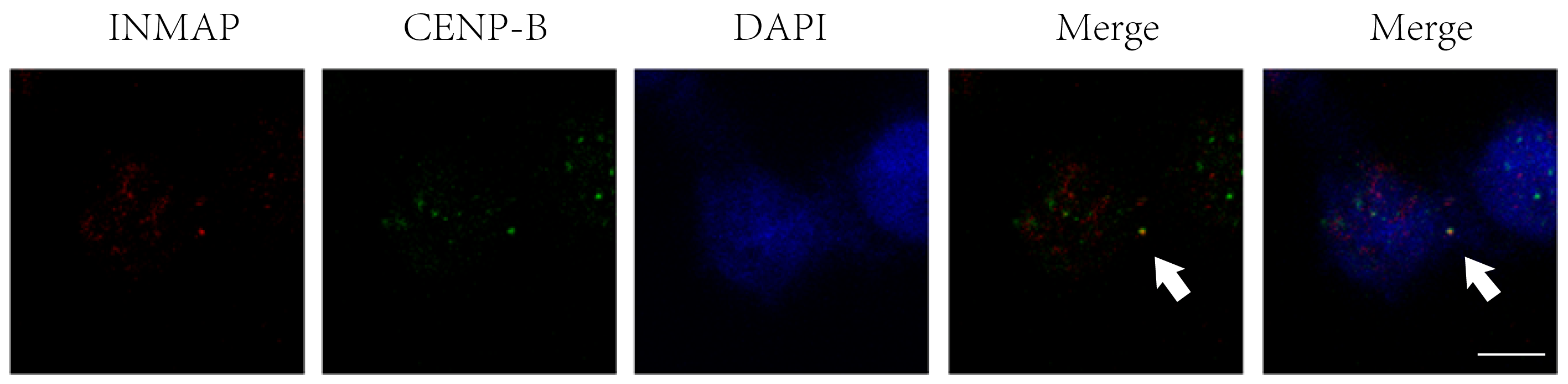

Supplement: Figure S1 — Close correlation between INMAP and CENP-B under a certain circumstances. Sub-cellular localization of INMAP and CNNP-B in HeLa cell nucleus was analyzed with anti-INMAP (red), anti-CENP-B (green) monoclonal antibodies and DAPI (blue). INMAP focus can overlap centromere in some circumstances, marked by the typical centromere protein CENP-B as the arrows indicated. Bar represents 10 μm. (TIF) [file pone.0091937.s001.tif]

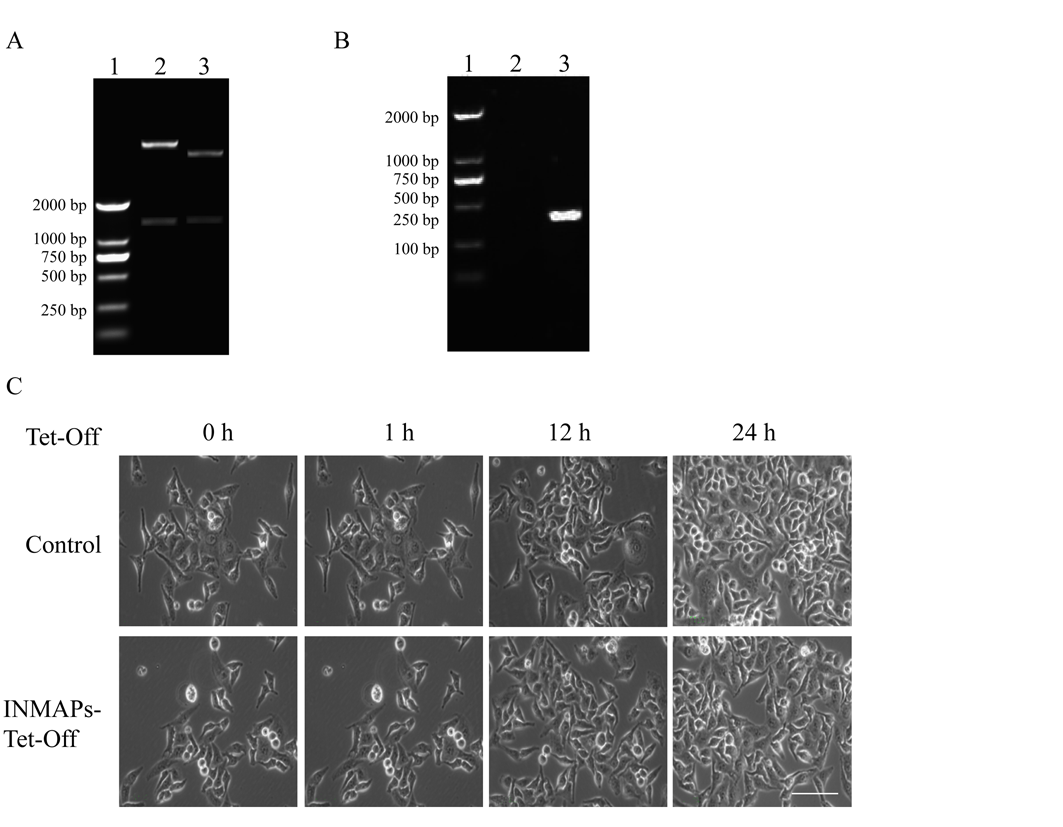

Supplement: Figure S2 — pTRE-hyg-INMAP (-) vector construction. A. The analysis of pTRE-INMAP (-) and pTRE-hyg-INMAP (-) double restriction enzyme digestion. Lane 1: DNA markers. Lane 2: pTRE-hyg-INMAP (-) double restriction enzyme digestion. Lane 3: pTRE-INMAP (-) double restriction enzyme digestion. B. DNA was extracted from INMAPs-Tet-Off cells and hygromycin gene was detected by PCR. Lane 1: DNA markers. Lane 2: Amplifying hygromycin from the HeLa cell genome by PCR. Lane3: Amplifying hygromycin from INMAPs-Tet-Off cells by PCR. C. Control and INMAPs-Tet-Off cells were cultured in tetracycline-free medium for indicated hours and photographed under a phase contrast microscope at 40× objective lens. Bars represent 50 μm. (TIF) [file pone.0091937.s002.tif]

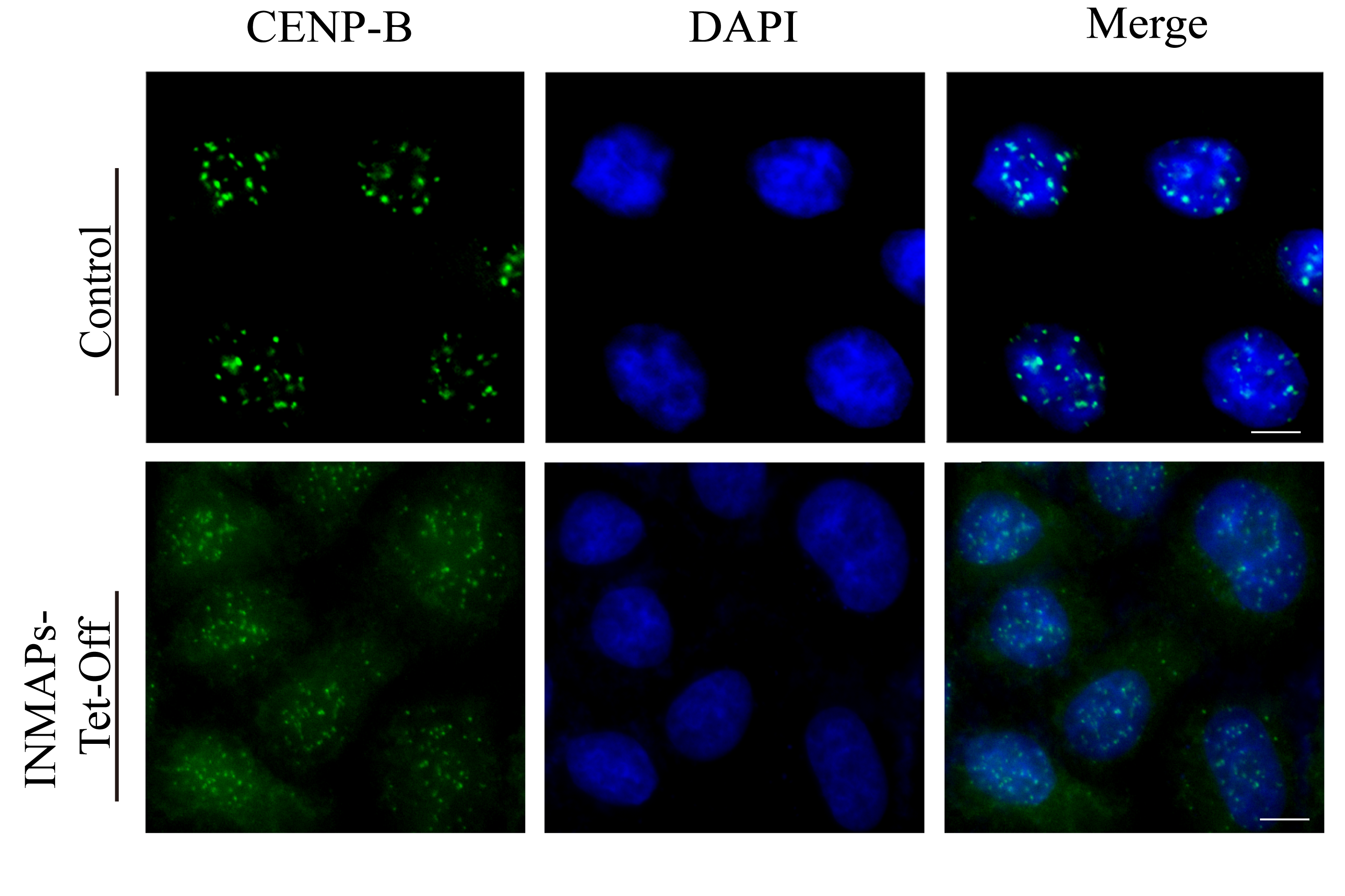

Supplement: Figure S3 — INMAPs-Tet-Off cells showed distinct halo-like staining of CENP-B. Centromere structure was analyzed with anti-CENP-B monoclonal antibody (green) and DAPI (blue) in HeLa and INMAPs-Tet-Off cells. INMAPs-Tet-Off cells had a halo like structure around the centromeres (C-halo). Under the same photographing condition, the clear doted CENP-B signals appeared in control, but “haloes” in the experimental group with diffused background. Bars represent 10 μm. (TIF) [file pone.0091937.s003.tif]

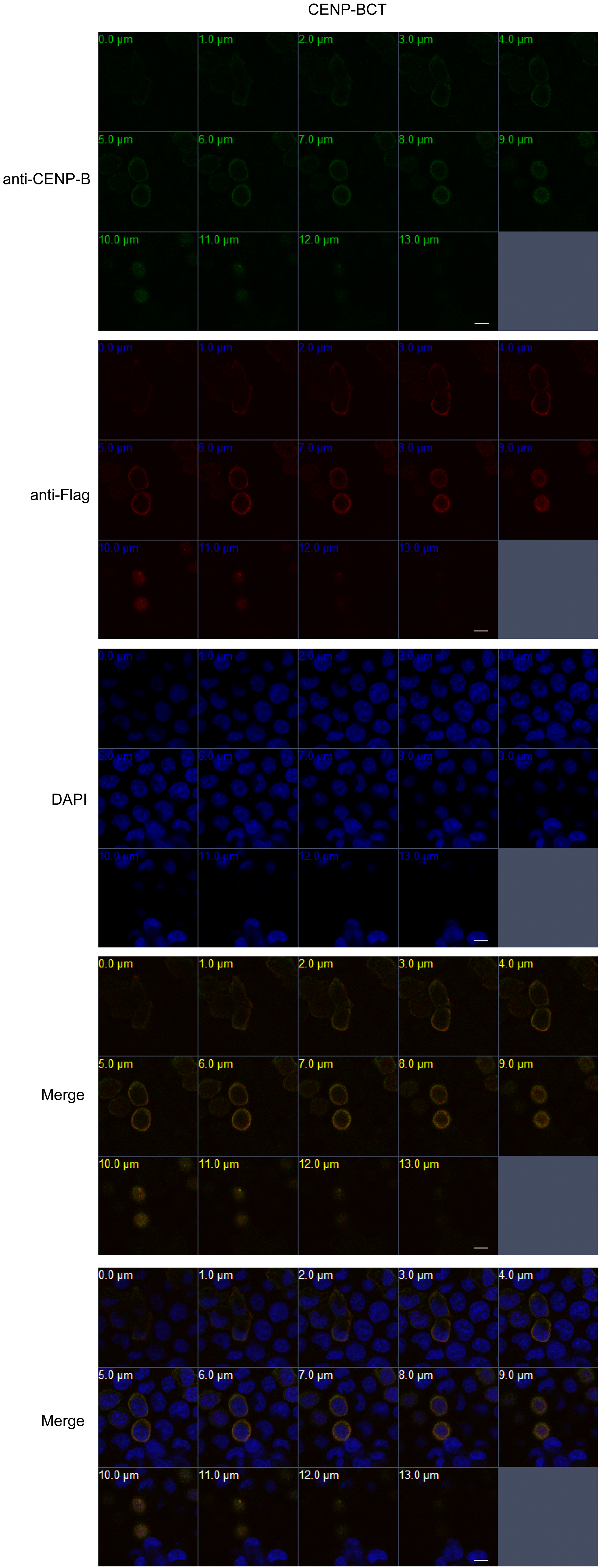

Supplement: Figure S4 — Scanning various cellular stacks of CENP-BCT cells with laser scanning confocal microscope. Transient transfection of the truncated CENP-B variant lacking the DNA-binding domain and putative DNA-binding domain (Flag-CENP-BCT) was conducted in HeLa cells and analyzed with anti-Flag (red), anti-CENP-B (green) monoclonal antibodies and DAPI (blue) by laser scanning confocal microscope, bars represent 10 μm. Interval of two stacks was 1 μm. (TIF) [file pone.0091937.s004.tif]

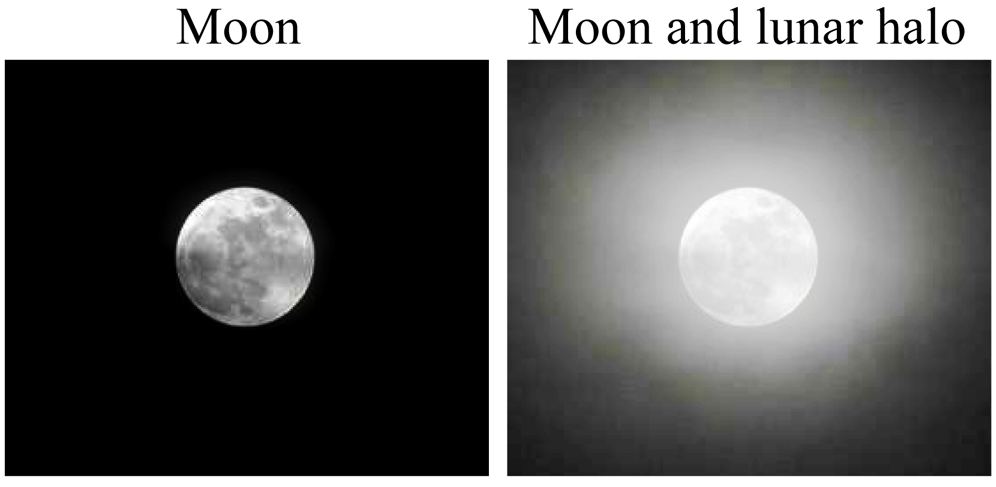

Supplement: Figure S5 — The moon in the sky at a clear night and a cloudy night. C-halo is like the lunar halo in the night. Left: the moon appears in the sky at a clear night without clouds or wind. A clear outline of the moon can be observed. This situation is analogous to the centromere in the normal nucleus. Right: the moon appears in the sky at a cloudy or windy night. Many halo-like clouds or fog appears near the moon. This situation predicts a rainy or foggy subsequent day. The centromere in the INMAPs-Tet-Off cells is similar to this type of moon, and CENP-B fluorescent signal is similar to the lunar halo around the centromere. The halo also predicts some non-dramatic events in the cell, e.g., the cell grows slowly. (TIF) [file pone.0091937.s005.tif]
